# The Use of Protein-Based Biomarkers for the Diagnosis of Cystic Tumors of the Pancreas

**DOI:** 10.1155/2011/413646

**Published:** 2011-10-24

**Authors:** Richard S. Kwon, Diane M. Simeone

**Affiliations:** ^1^Department of Internal Medicine, University of Michigan, 1500 E. Medical Center Drive, Taubman 3912, Ann Arbor, MI 48109-5362, USA; ^2^Departments of Surgery and Molecular and Integrative Physiology, University of Michigan, 1500 E. Medical Center Drive, Taubman 2210B, Ann Arbor, MI 48109-5343, USA

## Abstract

Proteomics is a powerful method used to identify, characterize, and quantify proteins within biologic samples. Pancreatic cystic neoplasms are a common clinical entity and represent a diagnostic and management challenge due to difficulties in accurately diagnosing cystic lesions with malignant potential and assessing the risk of malignant degeneration. Currently, cytology and other biomarkers in cyst fluid have had limited success in accurately distinguishing both the type of cystic neoplasm and the presence of malignancy. Emerging data suggests that the use of protein-based biomarkers may have greater utility in helping clinicians correctly diagnose the type of cyst and to identify which cystic neoplasms are malignant. Several candidate proteins have been identified within pancreatic cystic neoplasms as potential biomarkers. Future studies will be needed to validate these findings and move these biomarkers into the clinical setting.

## 1. Background

Pancreatic cysts are increasing in prevalence as cross-sectional imaging has become widely utilized. In recent population-based studies using magnetic resonance imaging (MRI) [1–4] and computerized tomography (CT) scans [5, 6], the estimated prevalence of cystic lesions ranges from 2.6% to as high as 44.7%. An autopsy study of 300 patients from Japan reported a prevalence of 24.3% [7]. Not surprisingly, the number of evaluations for these lesions is increasing [8, 9]. Management of these increasingly prevalent lesions can utilize a significant amount of health care resources in the form of diagnostic studies and surgical resections. Therefore, developing an accurate and cost-effective diagnostic test to assist in patient management is a clear priority.

This paper will focus on cystic neoplasms which are distinguished by the presence of mucinous or nonmucinous epithelium. Ninety percent of all cystic neoplasms are comprised of serous cystadenomas (SCAs), a non-mucinous lesion, as well as mucin-producing cystic tumors comprised of mucinous cystic neoplasms (MCNs) and intraductal papillary mucinous neoplasms (IPMNs) [10]. Rare cystic neoplasms include solid pseudopapillary lesions, lymphoepithelial cysts, and cystic degeneration of pancreatic ductal adenocarcinoma or neuroendocrine tumors (see Table 1). The most common nonneoplastic cyst is a pancreatic pseudocyst which is associated with acute pancreatitis and has no epithelium.

SCAs are characterized by bland cuboidal glycogen-rich epithelium. They tend to occur predominantly in women (87%) with a median age in the early 50s [11, 12]. SCAs are usually comprised of microcystic components, with a classic honeycomb appearance, though they can be macrocystic in appearance [12]. Up to 30% of these lesions will have a characteristic central scar. Their malignant potential is considered so low that they are generally not resected unless symptomatic.

MCNs have columnar mucinous epithelium with surrounding ovarian stroma (defined as hypercellular spindle cell bundles that lay just beneath the epithelium and usually show positive staining for estrogen and progesterone receptors) [13, 14] and typically present as large solitary macrocystic lesions in the body or tail of the pancreas [15]. They are thought to be separate from the main pancreatic duct but can be connected in up to 20% of cases [14]. They occur almost exclusively in women (95–98%) [14] during the fourth or fifth decades of life [15]. The rate of malignancy ranges from 6–30% at the time of resection [15–17]. Risk factors for malignancy include older age, the presence of a mural nodule with the cyst, and cyst size >4 cm [14, 15, 17]. The five-year survival is 100% in patients with benign disease and 60% in those patients that develop invasive carcinoma [17, 18]. Recurrence appears to occur only with invasive disease [15, 19].

IPMNs are characterized by columnar papillary mucinous epithelium that involves the main pancreatic duct, the side branch ducts (SB-IPMN) or both (mixed IPMN). IPMNs tend to occur more frequently in the head of the pancreas than the body and tail. Males have a slightly higher predilection for IPMNs than females do. The risk of malignancy is much higher in main duct disease (mean 70%) than side-branch disease (mean 25%) [20]. The reported overall 5-year survival rate for resected noninvasive IPMN ranges from 77 to 100%, whereas 5-year survival rate for invasive IPMN ranges from 30% to 75% [21].

Recently, IPMNs have been categorized into four epithelial subtypes—gastric, intestinal, pancreaticobiliary, and oncocytic, based upon cell morphology and expression patterns of glycoproteins containing mucin (MUC) [22]. Combinations of epithelium subtypes may be present within an individual lesion and therefore each IPMN is classified by the dominant component [22]. Based upon recent studies, these categories may explain the clinical behavior of the different IPMN subtypes [23, 24]. Gastric-type IPMNs primarily are located in the side branches and express MUC5AC but not MUC1 or MUC2 [23]. These rarely undergo malignant transformation [23], but if they do, they develop into tubular adenocarcinomas, which have a survival that is almost as poor as ductal adenocarcinoma [24]. Intestinal-type IPMNs are located mainly in the main pancreatic duct and express both MUC2 and MUC5AC [22]. These have a high frequency of malignant transformation to colloid carcinoma, which has a better prognosis than ductal adenocarcinoma [23, 24]. When compared to the other subtypes of IPMN, pancreaticobiliary-type IPMN is noted to occur more frequently in women than men and at a later age (mean 69.2 y versus 60.3–65.6 y) [23]. By immunohistochemistry, they express MUC1 and MUC5AC [22, 23] and may progress to tubular adenocarcinoma [23, 24]. The oncocytic-type IPMNs also express MUC5AC and MUC1 [22]. These tend to develop in people at a younger age than the other IPMN subtypes [23]. While these can progress to malignancy (oncocytic adenocarcinoma), they tend to be noninvasive [23] and have better survival than ductal adenocarcinoma [23, 24]. 

To date, the subtypes of mucinous tumors (MCN and IPMN) can only reliably be distinguished by surgical pathology. There are no presurgical tests that distinguish these cyst types with a high level of accuracy. Moreover, the triggers or markers for malignant transformation are unknown and the timeline to transformation remains unclear. As such, our knowledge of the natural history of these lesions is still limited.

In 2006, International Consensus Guidelines were developed by a team of experts to define management of cystic mucinous neoplasms [20]. For cystic neoplasms, the decision to undergo surgical resection versus surveillance should be tempered by patient's wishes, comorbidities, life expectancy, and the risk of malignancy versus the risk of surgery. If the patient is an appropriate surgical candidate, the guidelines suggest resection of all MCNs and any IPMN which involve the main duct, or side branch IPMN lesions that are symptomatic, have a solid component, or are >3 cm in size [20]. They recommend yearly surveillance for lesions <10 mm, and surveillance every 6–12 months for lesions 10–20 mm and every 3–6 months for lesions >20 mm. The surveillance interval can be lengthened after two years of no change [20]. A retrospective study of 147 patients demonstrated that the algorithm proposed by the guidelines had a sensitivity and negative predictive value of 100% but a specificity of 23% [25]. Other studies have shown similar results [26–30]. The algorithm therefore seems reasonably sensitive to identify those who do not need surgery, but given the low specificity, there remains a fairly high resection rate for patients with benign disease [21].

The clinical challenges of managing patients with pancreatic cystic neoplasms have several layers of complexity. First, one must differentiate between mucinous and non-mucinous cysts. This differentiation is important because their clinical management is different. Non-mucinous lesions generally do not require follow-up, whereas because of their premalignant potential, mucinous neoplasms are either resected or monitored in a surveillance program. Second, once a mucinous lesion is identified, one should distinguish between MCN and IPMN (in particular focal SB-IPMN) since the former should be resected whereas the latter can be monitored. This can be a difficult task in part because MCNs occasionally have communication with the main duct which is sometimes difficult to accurately identify by cross-sectional imaging or EUS [10, 20]. Moreover, there is no preoperative test that can identify the characteristic ovarian stroma of MCNs [10], and even on surgical pathologic analysis, the stroma may not be uniformly present, particularly with malignant transformation [31]. Third, amongst the mucinous lesions, one must differentiate between those that have high-grade dysplasia and cancer and those that are benign in order to appropriately refer those patients to surgery who would most benefit from resection. This differentiation would allow more selective recommendation of surgical resection for those who truly need it. Finally, given that the natural history of these lesions remains to be clarified, one must be able to identify those lesions that will go onto malignant transformation. To date, no single test or tests adequately addresses these challenges and as such, a biomarker or set of biomarkers are needed in order to address all four of these challenges. 

**Figure 1:**
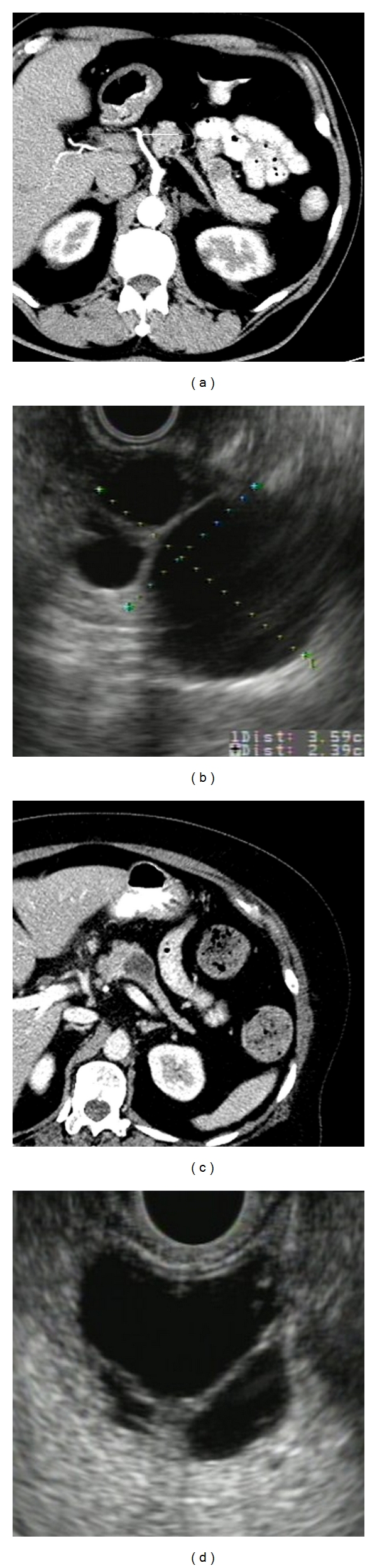
These images highlight the limitations of cross-sectional imaging and endoscopic ultrasound (EUS) in differentiating cyst types. By CT ((a)+(c)) and by EUS ((b)+(d)), the two cysts look very similar. The cyst in (a) and (b) was a macrocystic serous cystadenoma and the cyst in (c) and (d) was a mucinous cystadenoma. The histology of both was confirmed by surgical resection.

Current tests have limited ability to distinguish between mucinous and non-mucinous lesions or to identify malignant cysts (Figure 1). Cross-sectional radiologic imaging is limited in its ability to distinguish between the different types of cysts. The accuracy of CT and MRI to determine the correct histology ranges from 40–60% [32, 33]. New advances in CT and MRI technology that provide more detailed images may account for a modest increase in accuracy up to 84% [34]. Morphology by endoscopic ultrasound is also limited in its ability to distinguish between types of cystic tumors, with a sensitivity and specificity of 56% and 45%, respectively [35]. Furthermore, the accuracy of EUS morphology is limited by a lack of interobserver agreement [36]. 

Endoscopic ultrasound allows for fine needle aspiration of cyst fluid analysis. The tests of choice for diagnostic evaluation include cytology and carcinoembryonic antigen (CEA). Fluid cytology can be limited due to luminal contamination, highly variable amounts of extracellular mucin, and scant cellularity within the cyst [37]. The overall accuracy of cytology in identifying mucinous lesions is around 50% [35, 38]. There are no cytological findings which currently distinguish MCN from IPMN [39, 40]. Cytology has a specificity that approaches 100% but lower sensitivity in identifying the presence of malignancy [38, 41, 42]. To date, cyst fluid CEA remains the most accurate test to distinguish mucinous from non-mucinous cysts [35]. A prospective, multicenter study determined that a CEA >192 ng/mL had a 75% sensitivity and 85% specificity in distinguishing between mucinous and non-mucinous cysts. Its overall accuracy of 79% was higher than morphology, cytology, and other tumor markers previously identified in pancreatic cysts such as CA72-4 [43], CA19-9 [44], and CA 15-3 [35, 45]. However, fluid CEA is limited by the fact that there is broad overlap between types of lesions. In addition, this test is unable to distinguish between types of mucinous cysts, nor is there any correlation between elevated concentrations and risk of malignancy [46].

## 2. Biomarkers for Cystic Neoplasms

As a result of these current limitations, there has been considerable interest in finding other biomarkers that can better distinguish mucinous lesions and identify patients with tumors of higher malignant potential (i.e., with high-grade dysplasia or carcinoma) who would benefit most from surgical resection. The general approach that has been taken is to aspirate cyst fluid and use a variety of techniques to try to create highly sensitive and specific assays to identify subjects with high grade dysplasia or frank malignancy [47]. Pancreatic cyst fluid would appear to be an ideal source for a biomarker development due to its relative ease at obtainment by endoscopic ultrasound, which has a low complication rate when performed by an experienced endoscopist, and the presumed localization of relevant biological material from cyst epithelium [48]. 

There is a considerable interest in genetic material within cyst fluid and its potential as to serve as biomarkers. DNA mutations, such as K-ras, and allelic loss amplitude of a proprietary list of specific pancreatic cancer-related genes within cyst fluid have been studied as surrogate markers for mucinous and malignant cysts [49]. In a multicenter study of 113 patients, the authors reported that the presence of a cyst fluid K-ras mutation had a high specificity of 95% but low sensitivity of 45% for diagnosing mucinous cysts. The combination of a K-ras mutation followed by allelic loss showed a high specificity of 96% but a low sensitivity of 37% in diagnosing malignant cysts [49]. Subsequent studies have reported mixed correlation between these DNA mutations and final surgical pathology [50–52]. The added benefit over existing tests remains unclear, and as such, the role for DNA analysis will need to be clarified [46, 53]. 

MicroRNAs (miRNAs) are small (22 nucleotides) noncoding RNAs that regulate the stability and translation of mRNA transcripts. Deregulation of miRNA expression has been identified in several human cancers, including pancreatic adenocarcinoma [54–59]. Using a panel of 12 miRNAs that are upregulated in pancreatic cancer, Habbe et al. described the identification of abnormal miRNA expression in surgical histology from 15 noninvasive IPMNs compared to normal pancreatic tissue [60]. Moreover, they established the feasibility of identifying miRNA in pancreatic juice. The two miRNAs with the highest expression, miR-21 and miR-155, both of which have been shown in laboratory studies to inhibit apoptosis, had higher expression in the IPMNs (6 of 10, 60%) than normal controls (0 of 5), though this did not reach statistical significance due to a small number of samples. There was also an increased frequency of miR-155 expression in IPMN lesions with pancreaticobiliary and intestinal epithelium. Further studies will be required to validate these findings and to define the true utility of using miRNAs as biomarkers in pancreatic cyst fluid.

## 3. Protein-Based Biomarker Strategies

Another strategy for biomarker development is to identify specific proteins already known to be involved in pancreatic cancer. One group used multiplex assays with 54 proteins associated with pancreatic cancer to demonstrate differential protein expression between noninvasive IPMN and SCA (34 out of 51 proteins, 67%) and noninvasive MCN and SCA (13 out of 51 proteins, 25%) [61]. When using a panel of 14 proteins, the accuracy of distinguishing IPMNs from SCAs was 92% [61]. Another group took a more specific approach and examined the role of Prostaglandin E(2), which they had shown to have increased expression in pancreatic cancer tissue over normal pancreatic tissue [62], in distinguishing between types of mucinous cysts. From fluid obtained from 58 resected cystic lesions, they demonstrated that cyst fluid PGE(2) concentrations were greater in IPMNs versus MCNs (2.2 ± 0.6 versus 0.2 ± 0.1 pg/mol, *P* < 0.05) and that the mean level of PGE(2) increased with the degree of dysplasia in IPMN lesions [63]. However, there was noted to be an overlap in PGE(2) concentrations in benign MCNs (*n* = 11) and SCAs (9*n* = 5), thereby limiting the utility of this biomarker in the clinical setting. These studies demonstrate that targeting proteins associated with pancreatic cancer show potential in identifying appropriate biomarkers for cystic lesions and will require further investigation and validation.

As described above, immunohistochemistry of surgical pathology has demonstrated differential mucin profiles in IPMN [64, 65] and it would seem logical to expect mucin profiles in cyst fluid to identify lesions at risk for malignancy. A recent study has demonstrated differential mucin expression in cyst fluid from 40 surgically resected IPMNs using enzyme-linked immunosorbent assays [66]. Patients with high grade dysplasia or carcinoma (*n* = 19) were categorized as “high risk.” Cyst fluids MUC2 and MUC4 were elevated in high-risk patients as compared to low risk patients (10 ± 3.0 ng/mL versus 4.4 ± 1.2 ng/mL, *P* = 0.03; 20.06 ± 10.6 ng/mL versus 4.5 ± 1.4 ng/mL, *P* = 0.03, resp.). There was no difference in MUC1 or MUC5AC concentrations between the two groups. Cysts with gastric epithelium (*n* = 23) had statistically significant lower expressions of MUC2, MUC4, and MUC5AC compared to pancreatic cystic tumors without gastric epithelium. Cysts with intestinal epithelium (*n* = 8) had statistically significant higher elevations of MUC2 compared to nonintestinal cysts and a trend towards higher MUC4 concentrations. There was no discernible difference in MUC concentrations in pancreaticobiliary epithelium cysts compared to those without pancreaticobiliary epithelium. These findings have not yet been validated; however, this study highlights the potential for risk stratification based upon MUC expression in cyst fluid.

Other proteins previously identified in pancreatic cancer have also been studied to more accurately identify IPMN harboring malignancy. Mutant K-*ras* protein has been identified in cyst fluid using mass spectrometry [67]. The expression of Plectin-1, a marker found to be increased in ductal adenocarcinoma, has also been identified in fluid from malignant mucinous cysts [68]. Cytokine IL-1*β* is markedly elevated in high-risk patients compared to low risk patients in a small number of IPMNs [69]. These findings have yet to be validated and are thus far experimental in nature.

## 4. Proteomics of Cyst Fluid

Proteomics is an attractive method for identifying proteins within the cyst fluid which can differentiate mucinous cysts or identify malignancy with greater accuracy. Traditionally, proteins are separated by two-dimensional gel electrophoresis with subsequent mass spectrometric identification of protein spots or by protein digestion and mass spectrometric identification of peptide sequences. Proteomics overcomes the shortcoming of using DNA or mRNA analysis, whose changes may not reflect actual protein expression [70, 71] or include posttranslational modifications. Furthermore, proteomic analysis may provide information on the pathogenesis of these lesions.

The challenge of using proteomics is the complexity of the proteome. The method can identify a large number of proteins but interpretation of the results may be clouded by the signal of the most abundant proteins, and thus proteins present in very small concentrations may not be easy to identify. Within the pancreatic cyst fluid itself, the vast variety of proteins are a reflection of cyst epithelium, luminal contamination (if fluid is obtained by transgastric or transduodenal aspiration), plasma proteins, mucus, and possibly pancreatic enzymes, if there is a connection of the cyst to the pancreatic ductal system. Protein concentration yield may be subject to degradation by endogenous peptidases [72] or post-translational modifications [73]. Proteomics has been used to successfully identify potential biomarkers in the tissue [74, 75], serum [76], and pancreatic juice [77, 78] of patients with pancreatic ductal adenocarcinoma. A method to perform proteomic analysis using paraffin-embedded archival slides of a noninvasive IPMN carcinoma-in-situ has also been described [79]. 

Interest in using proteomics in pancreatic cyst fluid analysis is growing. The feasibility of proteomic analysis of pancreatic cyst fluid was established by Scarlett et al. [80]. In this proof of concept study, cyst fluids from 10 patients (including 3 ductal adenocarcinomas, 2 mucinous cystadenoma, and 1 IPMN) were analyzed using SELDI-TOF mass spectrometry. Reproducible protein profiles were demonstrated amongst the adenocarcinoma patients with differential expression in twelve protein peaks identified. These findings suggest that proteomics is a viable method for identifying potential biomarkers within cyst fluid.

Two recent studies advanced the use of proteomic analysis to identify biomarkers in cyst fluid. Ke et al. used small volumes (<40 *μ*L) from EUS fine needle aspirates and grouped patients according to their cytology results ((a) benign: no evidence of benign mucinous epithelium, atypical cells or carcinoma; (b) benign mucinous epithelium; (c) atypical/suspicious; (d) malignant) [81]. Fluid was analyzed using MALDI-TOF mass spectrometry with LC/MS/MS protein identification, 2D gel electrophoresis, or GeLC/MS/MS (tryptic digestion of proteins fractionated by SDS-PAGE and identified by LC/MS/MS). The first two techniques proved to be unsatisfactory, presumably from endogenous peptidases which splintered native proteins in numerous locations [81]. Mass spectrometry yielded homologs within three families of proteins associated with pancreatic cancer, including mucins, CEACAMs, and S100s. The authors conclude that LC/MS/MS mass spectrometry provides useful information on biomarkers within cyst fluid using small volumes of fluid.

The same technique was used by Cuoghi et al. in a study of 8 patients who underwent surgical resection for symptomatic pancreatic neoplasms. Fluid was aspirated directly from the surgical specimens, thereby avoiding potential gastrointestinal luminal contamination. Proteins were separated by SDS-PAGE and then analyzed by LC/MS/MS. The total number of proteins identified in the cyst samples ranged from 220 to 727. They identified 38 proteins unique to neuroendocrine tumors, 18 unique to serous cystadenomas, 92 unique to MCNs, and 29 unique to IPMNs. Analysis of known proteins revealed that several proteins identified in the mucinous lesions (MCNs and IPMNs) were previously reported to be upregulated pancreatic cancer-associated proteins. The findings were confirmed by immunohistochemistry for two of the identified proteins, olfactomedin-4 (OLFM4) and the cell surface glycoprotein MUC18. Clearly, proteomics shows great promise in identifying potential biomarkers (see Table 2). Further studies and refinement of technique will hopefully yield reliable candidate biomarkers that can be validated in clinical studies.

## 5. Glycoproteomics

Glycoproteomics specifically examines carbohydrate modification or glycosylation of proteins. Aberrant glycosylation is a hallmark for tumorigenesis and tumor progression and not surprisingly, many previously identified biomarkers are glycoproteins. The advantage of glycoproteomics is the focused isolation of glycoproteins by specific binding of glycosylation sites. This specificity reduces the complexity of sample protein populations. As such, this method significantly increases the detection sensitivity for low abundance proteins [84]. Analytic approaches have been broadly categorized as glycoprotein-based analysis or glycopeptide-based analysis [84, 85]. The former begins with enrichment of glycoproteins using lectin and separation techniques to enrich the protein fractions. The latter uses glycopeptides that are digested and then deglycosylated. Peptide identification is then performed by mass spectrometry.

Glycoproteomics has already shown promise as a biomarker development tool in pancreatic cancer. A technique using lectin affinity chromatography, liquid separation, and characterization by mass spectrometry was demonstrated in serum of patients with pancreatic cancer [85]. Sialylated plasma protease C1 inhibitor was shown to be downregulated in cancer serum. Downregulation of the N83 glycosylation sites was also observed. Ninety-two individual glycosylation sites with 41 glycoproteins were identified and 202 glycan peaks with 104 unique carbohydrate structures were detected during glycan profiling using different separation techniques. Forty-five oligosaccharides were found altered in pancreatic cancer serum of which 44 were distinct in the cancer sample [85]. Based on these promising results, glycoprotein microarrays have been created as a high throughput tool to differentiate serum samples from patients with pancreatic cancer, from chronic pancreatitis and normal subjects [86, 87].

This approach has been expanded to the use of glycoproteomics in cystic neoplasms. Using a novel antibody-lectin sandwich array (ALSA) that targets glycan moieties on proteins [88], Haab et al. measured protein expression and glycosylation of MUC1, MUC5AC, MUC16, CEA, and other proteins associated with pancreatic cancer in 53 cyst fluid samples from surgically resected lesions (17 MCN, 15 IPMN, 15 SCA, and 9 pseudocysts) [82]. Wheat germ agglutination of MUC5AC was markedly elevated in MCN and IPMN but not SCAs or pseudocysts. CA19-9 could distinguish between MCN and IPMN with a sensitivity and specificity of 82% and 93%, respectively. MUC1 was elevated in serous lesions compared to pseudocysts and mucinous cysts. MUC5AC in combination with CA19-9 (sensitivity 87%, specificity 86%) outperformed fluid CEA (37% sensitivity, 80% specificity) in distinguishing mucinous from nonmucinous cysts. This study shows that glycan variants of proteins within cyst fluid may prove to be useful biomarkers and highlights an area warranting further evaluation. Validation studies are currently in progress. In addition, it remains to be determined if this approach will be useful in separating malignant and non-malignant lesions.

## 6. Conclusions

The clinical management of pancreatic cystic neoplasms is difficult due to the lack of sufficiently sensitive and specific diagnostic tests to differentiate cyst types and the presence of malignancy. Pancreatic cyst fluid provides an appealing source for improved biomarker development, particularly by proteomic analysis. Preliminary work with cyst fluid glycosylated mucins show promise in distinguishing mucinous from non-mucinous cysts and differentiating types of mucinous cysts. Cyst fluid homologs of mucin, CEACAMs and S100s, and other proteins associated with pancreatic tumorigenesis have been identified as potential biomarkers for malignancy within cyst fluid. These results will all need to be studied and validated in larger more adequately sized test and training sets of pancreatic cyst fluid for full biomarker development. Given that the field is currently limited by the lack of adequate numbers of pancreatic cyst fluid samples for analysis, it will be important that resources for fluid samples are further developed. As we close our gaps in knowledge regarding natural history of mucinous cysts and the relationship between epithelial subtypes and prognosis, biomarkers will likely play a prominent role in the management of cystic neoplasms.

## Figures and Tables

**Table 1:** Types of pancreatic cystic neoplasms.

Mucinous
Mucinous cystic neoplasm
Intraductal papillary mucinous neoplasm (IPMN)
Non-mucinous
Serous cystadenoma
Solid pseudopapillary neoplasm
Lymphoepithelial cysts
Cystic degeneration of ductal adenocarcinoma
Cystic neuroendocrine tumor
Cystic acinar cell carcinoma

**Table 2:** Potential biomarkers identified to date in pancreatic cyst fluid.

Genetic biomarkers	References
DNA-based	
K-ras	[49]
Allelic loss amplitude	[49]
RNA-based	
miR-21	[60]
miR-155	[60]

Protein-based biomarkers	

Prostaglandin E(2)	[63]
Interleukin-1*β*	[69]
MUC1	[81, 82]
MUC2	[66]
MUC4	[66]
MUC5AC	[81–83]
MUC5B	[81]
MUC6	[81, 83]
MUC16	[81]
MUC18	[83]
CA 19-9	[82]
Plectin-1	[68]
S100-A6, 8, 9, 11	[81]
CEACAM 1, 5, 6, 7	[81]
BGP-1	[83]
Tspan-8, 27, 28	[83]
CD55	[83]
E-cad	[83]
Glutathione S-transferase P	[83]
Olfactomedin-4	[83]
Prostate stem cell antigen	[83]
Pyruvate kinase isozymes M1/M2	[83]
Ras-related protein Rab-8A	[83]
Rho-related GTP-binding protein RhoC	[83]
Trefoil factor 1,2	[83]
VE-cadherin	[83]
Protein-Z-dependent protease inhibitor	[83]
von Willebrand antigen 2	[83]
